# Double-Layered Patella (DLP) in Multiple Epiphyseal Dysplasia (MED)

**DOI:** 10.5334/jbr-btr.1219

**Published:** 2017-02-02

**Authors:** Annemieke Milants, Michel De Maeseneer, Johan De Mey

**Affiliations:** 1 UZ Brussel, BE

**Keywords:** Double Layered Patella (DLP), Multiple Epiphyseal Dysplasia (MED), multipartite, patella, anterior knee pain

## Abstract

Double-layered patella (DLP) is a rare form of bipartite patella, pathognomonic for a certain type of chondrodysplasia, named multiple epiphyseal dysplasia (MED). This patellar deformity may be asymptomatic, but it may also cause several complaints, including anterior knee pain and severe maltracking of the patella. We present the case of a young man with recurrent anterior knee pain, mainly provoked by movement, who was already known to have MED.

## Introduction

Multiple epiphyseal dysplasia (MED) is a type of chondrodysplasia, with both autosomal dominant and recessive inheritance, depending on the affected gene [2,3,4]. It causes delayed ossification of growth centers in tubular bones, an irregular and fragmented appearance when these ephiphyses begin to ossify, a higher incidence of coxa vara and slipped capital femoral head in children, dysplastic epiphyses, and eventually premature osteoarthritic changes. The hips and knees tend to be the most affected [3,4].

The double-layered patella (DLP) was first described by Buttner in 1925 and is now considered a pathognomonic sign for multiple epiphyseal dysplasia (MED) [1,3]. DLP, as the name suggests, consists of two patellar segments, anterior and posterior, which are divided by a coronal septum. It can be present in both the dominant and recessive form of MED. It is sometimes asymptomatic, but it may also cause a variety of clinical problems, including anterior knee pain.

## Case Report

**Figure 1:**
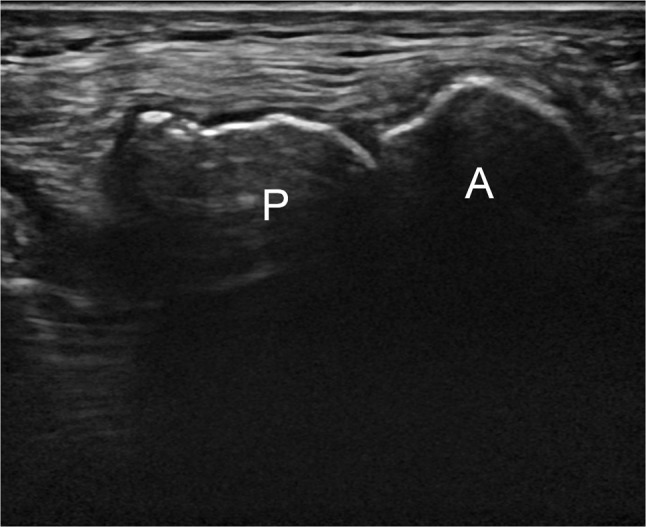
Ultrasound image of the patella, transverse viewpoint. Two patellar segments are visible: an anterior (A) and a posterior (P) segment.

**Figure 2:**
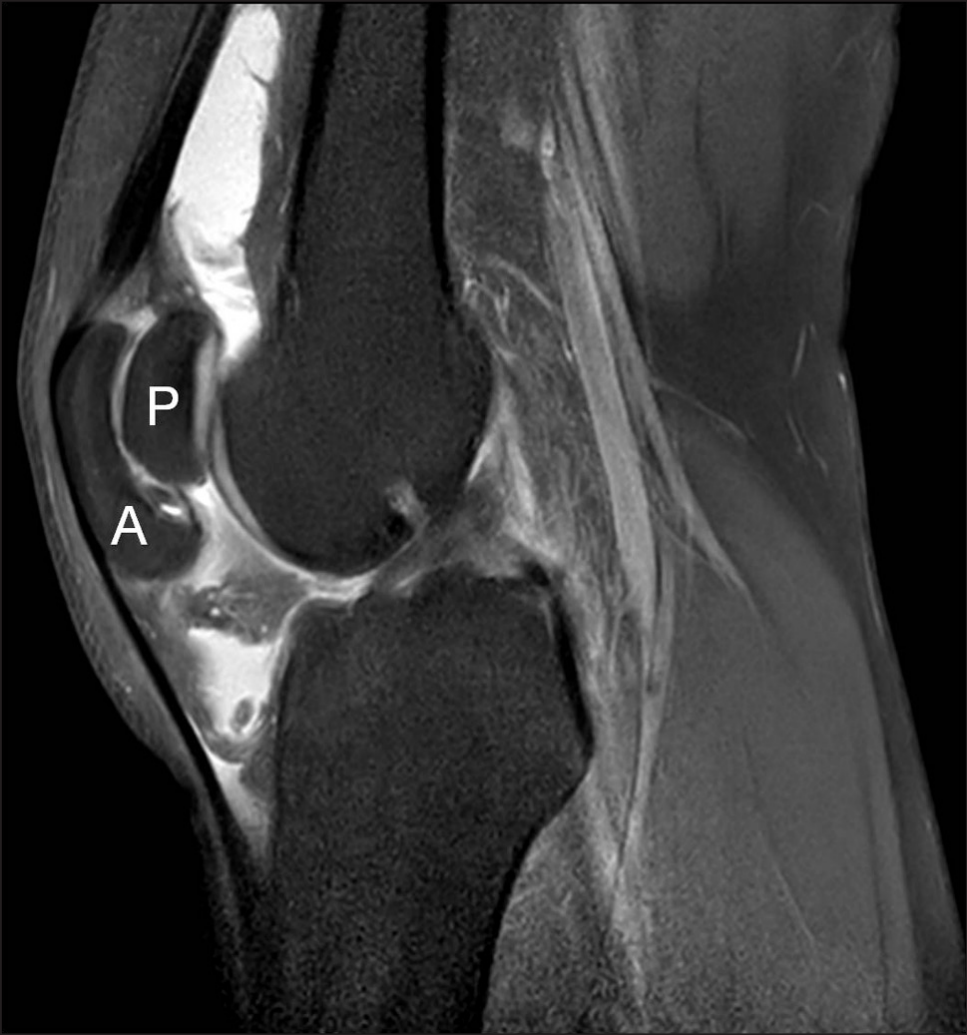
Sagittal PD fat-sat image confirming the double-layered patella. This image also demonstrates a significant intra-articular fluid effusion, as well as some fluid in the Hoffa patpad. A: anterior patellar segment; P: posterior patellar segment.

**Figure 3:**
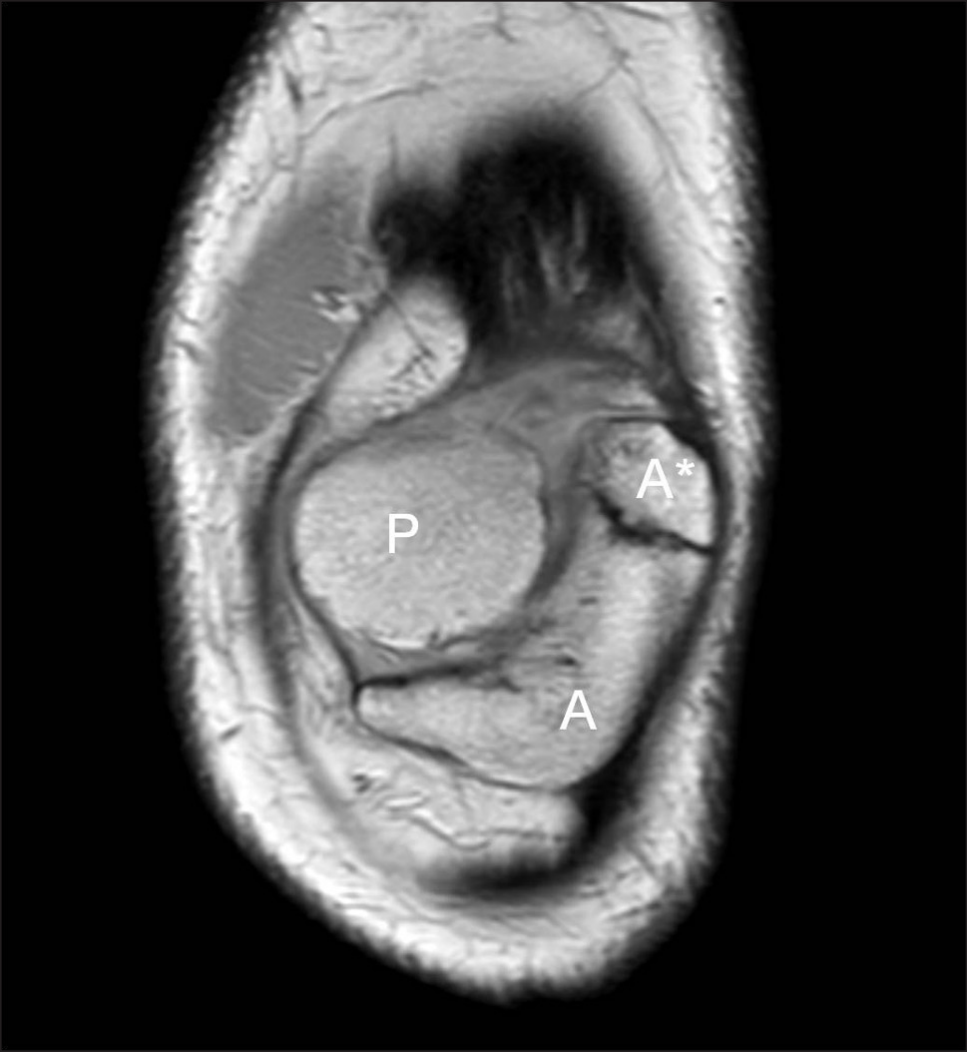
Coronal PD image demonstrating the bipartite character of the anterior patellar segment (A and A*). This coronal section also shows the posterior patellar segment (P).

**Figure 4:**
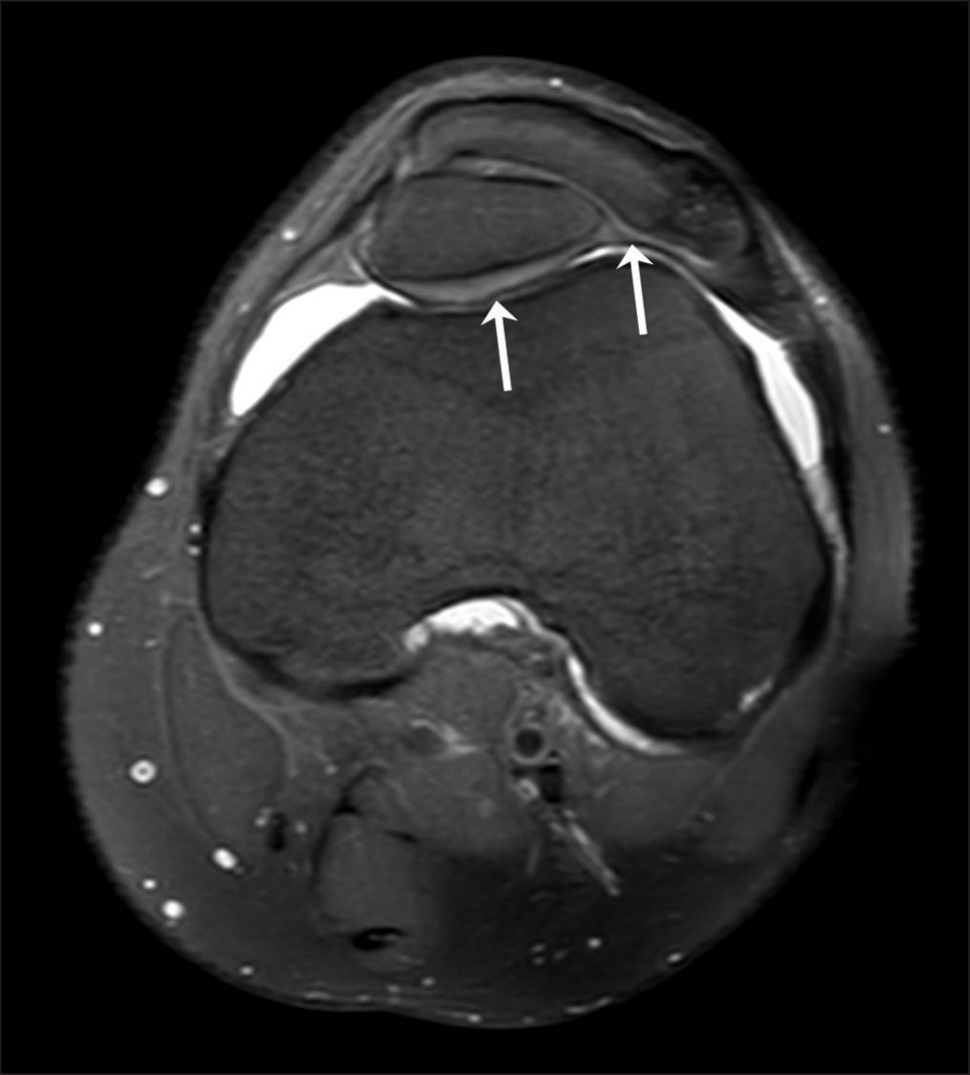
Axial PD fat-sat image demonstrating that both the anterior and posterior patellar segment have their own cartilage layer (arrows).

**Figure 5:**
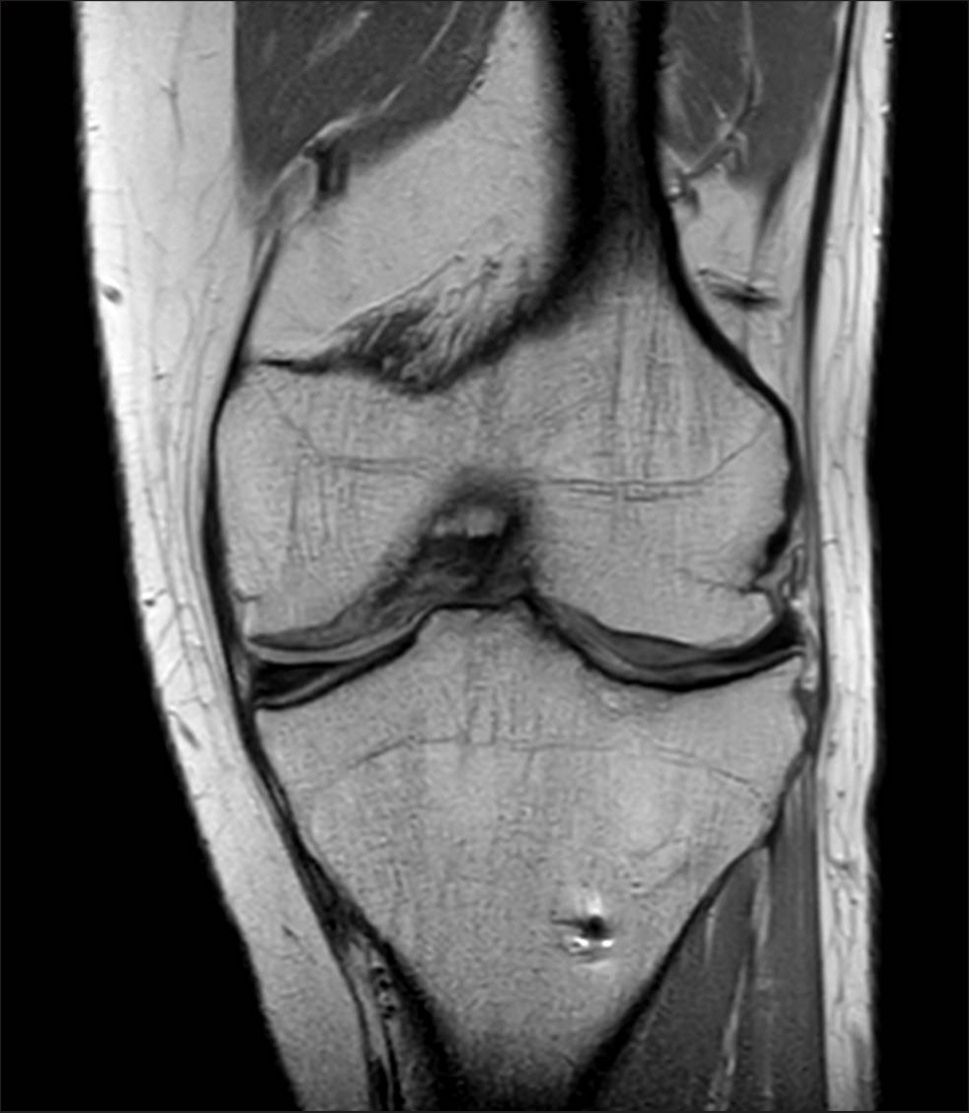
Coronal PD image demonstrating a dysplastic configuration of the femoral condyles and lateral and medial tibial plateau.

A 26-year-old man presented for ultrasound examination of anterior knee pain, which usually presented after physical effort and movement of the knee. The diagnosis of MED had already been made in the past, but he showed no striking physical features of chondrodysplasia. No standard radiograph was taken. Ultrasound showed a moderate effusion in the knee joint and some fluid in Hoffa’s fat pad. There was also some tendinosis of the quadriceps insertion. In the axial plane, two patellar segments were clearly visible (Figure 1). An MRI was performed a month later and confirmed the DLP (Figure 2), of which the anterior patellar segment showed a bipartite configuration of its own: type 3 bipartite patella with the smaller fragment on the superolateral side (Figure 3). Both the anterior and posterior segment possessed a cartilage layer (Figure 4). There was also a clearly dysplastic aspect of the distal femur and tibial plateau (Figure 5).

## Discussion

**Figure 6:**
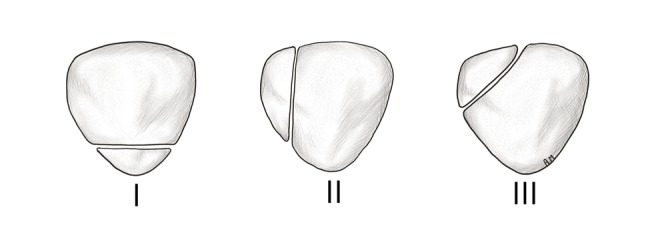
Sketch illustrating the three main types of bipartite patella, according to Eric Saupe.

Bipartite patella is known as a developmental anomaly and is usually regarded as a variant of normal ossification. It is seen in about 2 percent of the population and is more common in males (male-to-female ratio is 9: 1). It is bilateral in most patients and usually asymptomatic. It can, however, also be a cause of anterior knee pain [5]. Eric Saupe described the most frequent types of bipartite patella (Figure 6). In type 1, the patella is divided by a transverse split in the lower third. In type 2, there is a longitudinal split which divides the patella in an outer quarter bone fragment and an inner three-quarter bone fragment. In type 3, there is an oblique split which divides the patella into a larger inferomedial fragment and a smaller superolateral fragment [6].

DLP, however, is another, more rare form of bipartite patella. It was first described by Buttner in 1925 and is now considered as a pathognomonic sign for multiple epiphyseal dysplasia (MED) [1,3]. It consists of two layered patellar segments with a coronal septum dividing them [1,3,4]. The anterior segment is embedded in the quadriceps and patellar tendons, and it possesses its own layer of hyaline cartilage facing the posterior segment. The posterior segment forms the articulating surface facing the trochlea [1]. DLP may cause a variety of clinical problems, including anterior knee pain, clicking or locking, and patellar dislocation [2,3]. Often there is a delayed and painful movement of the posterior patellar segment that has no tendinous insertions, which causes a painful snapping of the patella [1].

When symptomatic, a surgical intervention should be considered. Fusion of the two patellar parts appears to be the most successful method of treatment, safeguarding the function of both the anterior and the posterior segment [1,3]. Another option is the resection of either the anterior or the posterior segment [1].

Usually, the first imaging technique is the traditional X-ray, which will clearly depict both patellar segments. Note that an isolated, incomplete form of DLP may exist. For this reason, an axial patellar view is recommended [2]. Classical X-ray will also demonstrate the epiphyseal deformities of the femoral condyles and tibial plateau, which is typical in MED. MRI will demonstrate the anomalies in more detail, giving more information about the tendinous insertions, patellar cartilage layers, epiphyseal dysplasia, and potential osteoarthritic changes.
